# Tumor Characterization Using [^18^F]FDG PET Radiomics in a PD-L1-Positive NSCLC Cohort

**DOI:** 10.3390/ph19010103

**Published:** 2026-01-07

**Authors:** Bernadett Erzsébet Kálmán, Agnieszka Bos-Liedke, Dániel Dezső, Ewelina Kaminska, Mateusz Matusewicz, Ferenc Budán, Domokos Mathe, János Girán, Dávid Sipos, Éva Pusztai, Árpád Boronkai, Zsombor Ritter

**Affiliations:** 1 Department of Medical Imaging, Medical School, University of Pécs, 7621 Pécs, Hungary; dezso.daniel@pte.hu (D.D.); ritterzsombor@gmail.com (Z.R.); 2 Department of Biomedical Physics, Adam Mickiewicz University, Uniwersytetu Poznanskiego 2, 61-614 Poznan, Poland; agnbos@amu.edu.pl; 3 HealthQuest Sp. z o.o. Adama Mickiewicza 63, 01-625 Warszawa, Poland; 4 Faculty of Economics, University of Life Science, 60-637 Poznan, Poland; mateusz.matusewicz16@gmail.com; 5 Institute of Physiology, Medical School, University of Pécs, 7624 Pécs, Hungary; budan.ferenc@pte.hu; 6 Department of Biophysics and Radiation Biology, Semmelweis University, 1094 Budapest, Hungary; mathe.domokos@semmelweis.hu; 7 Department of Public Health Medicine, Medical School, University of Pécs, Szigeti Street 12, 7624 Pécs, Hungary; janos.giran@aok.pte.hu; 8 Department of Medical Imaging, Faculty of Health Sciences, University of Pécs, 7621 Pécs, Hungary; david.sipos@etk.pte.hu; 9 Department of Oncotherapy, University of Pécs Clinical Center, Édesanyák Street 17, 7624 Pécs, Hungary; pusztai.pulmoonco@gmail.com (É.P.); arpad.boronkai@gmail.com (Á.B.)

**Keywords:** stage III, NSCLC, FDG, PET-CT, PD-L1, radiomics, durvalumab, NLR

## Abstract

**Background**: Durvalumab consolidation following radiochemotherapy is now the standard treatment for unresectable stage III non-small cell lung cancer (NSCLC). [^18^F]FDG PET/CT offers valuable insights not just for staging but also for tumor characterization via radiomics, which can potentially predict histology, immunophenotype, and prognosis. **Methods**: We conducted a retrospective analysis of [^18^F]FDG PET/CT scans from stage IIIA–IIIB NSCLC patients treated at the Clinical Centre, University of Pécs. All biopsy samples were classified histologically (squamous vs. adenocarcinoma) and tested for PD-L1. Lung tumors were segmented using MEDISO InterViewTM FUSION software (version 3.12.002.0000). with an SUVmax threshold of four. Imaging features were extracted and compared based on histology, PD-L1 status, and neutrophil-to-lymphocyte ratio (NLR)-based prognosis groups. Statistical analyses were performed with Jamovi (v2.6.44), using Shapiro–Wilk, *t*-test/ANOVA, Mann–Whitney/Kruskal–Wallis, or Chi-square tests as appropriate. **Results**: Fifty-six patients were included (38 PD-L1-positive, 18 -negative). Among PD-L1-positive cases, poor versus good NLR prognosis groups differed in maximum diameter (*p* = 0.046), short-zone emphasis (*p* = 0.026), and zone-length non-uniformity (*p* = 0.027). Focusing on PD-L1-positive squamous carcinoma, maximum diameter, metabolic tumor volume, busyness, and coarseness showed significant differences (all *p* < 0.05). SUVmax, mean SUV, SUVpeak, and complexity were higher in squamous than in adenocarcinoma subtypes. PD-L1-positive and -negative squamous tumors differed in zone percentage (*p* = 0.039) and long-zone high gray-level emphasis (*p* = 0.024), while no significant differences were observed among adenocarcinomas. **Conclusions**: [^18^F]FDG PET/CT radiomics showed potential for differentiating NSCLC histological subtypes and for identifying PD-L1-associated imaging patterns in squamous cell carcinoma. In addition, certain metabolic features were associated with NLR-based prognostic groups in PD-L1-positive patients.

## 1. Introduction

Non-small cell lung cancer (NSCLC) is the most common type of lung cancer, leading to significant illness and death worldwide [1,2]. In recent years, immunotherapy after concurrent or sequential radiochemotherapy has marked a breakthrough in treating patients with locally advanced, unresectable disease. Durvalumab (Imfinzi), a Programmed Death-Ligand 1 (PD-L1) monoclonal antibody, has become the standard of care for stage III NSCLC patients who show no disease progression following either concomitant or sequential radiochemotherapy, based on the results of the PACIFIC study series. To highlight in more detail stage III NSCLC is considered as a complex group of lung cancer patients. The TNM-9 classification offers a more detailed characterization of stage III disease [3]. Certain proportion of stage III/A patients can be candidate for surgery, even a few individually selected patients with stage III/B disease, but technical or medical inoperability affects a considerable rate of stage III disease. Curative approach of these patients can be concurrent or sequential chemoradiotherapy followed by consolidation durvalumab treatment, based on the results of PACIFIC and PACIFIC-6 clinical trials [4,5]. Subgroup analysis of the PACIFIC trial has proven significant DFS and OS advantage of patients receiving durvalumab compared to patients in the comparator (placebo) arm in the PD-L1-positive population. OS benefit was not found in patients with PD-L1 TC expression < 1% (median DFS: 5.5 vs. 24.9 months in the PD-L1 ≥ 1% group and 5.6 vs. 10.7 months in the PD-L1 < 1% group; median OS: 63.1 vs. 29.6 months in the PD-L1 ≥ 1% group and 33.1 vs. 43.0 months in the PD-L1 < 1% group).

Multiple clinical trials have demonstrated the benefit of neoadjuvant or perioperative immunotherapy (nivolumab, pembrolizumab, durvalumab, atezolizumab) over neoadjuvant chemotherapy in patients with stage I/B–III/B NSCLC. Event free survival (EFS) or disease-free survival (DFS) were the primary endpoints, for which pathological complete response (pCR) and major pathological response (MPR) are strong predictors. These trials included non-EGFR/ALK driven tumours. In the CheckMate 816 study, a favorable outcome was observed independent of PD-L1 expression level [6,7]. The goal of treatment is to eradicate residual microscopic tumor cells, lower the risk of local and distant progression, and enhance long-term overall survival [4,8]. The use of 2-deoxy-2-[^18^F]fluoro-D-glucose ([^18^F]FDG) Positron Emission Tomography combined with Computed Tomography (PET/CT) has become essential in modern imaging and cancer diagnosis. This imaging technique not only improves staging accuracy but also offers opportunities for examining histological and molecular differences, evaluating treatment response, and predicting outcomes [9,10,11,12,13]. Radiomics is a quantitative evaluation method of medical imaging to analyze the available data to underpin clinical decision-making. Elucidating the correspondence between histological and in vivo imaging-derived data, compared to the outcome of individualized therapies, may support the development of novel evidence-based therapeutic guidelines [14].

In our study, we retrospectively identified primary lung cancer sites on [^18^F]FDG PET/CT scans. All patients underwent tissue sampling and were tested for PD-L1 positivity. Our goal was to create a homogeneous patient cohort selected under consistently strict conditions, making the study more robust. We aimed to evaluate whether [^18^F]FDG PET radiomics could differentiate between patients with various histological subtypes or immunophenotypes (e.g., PD-L1 expression) and predict prognosis [15,16]. The prognostic stratification was assessed according to an approved clinical prognostic factor in PD-L1-positive patients: the neutrophil-to-lymphocyte ratio (NLR) [17,18,19].

## 2. Results

We examined a cohort of 56 NSCLC patients with stages IIIA–IIIB, including 38 PD-L1-positive and 18 -negative cases. The cohort comprised 30 males and 26 females. Patient clinicopathological features are detailed in Table 1.

Based on NLR values, PD-L1-positive patients were divided into good- or poor-prognosis groups (30 vs. 8), and their imaging features were compared. Non-parametric tests revealed significant differences in the following features: maximum diameter (mm) (*p* = 0.046; medians: 70.76 for poor vs. 57.58 for good); short-zone emphasis (*p* = 0.026; medians: 0.80 for poor vs. 0.86 for good); and zone-length non-uniformity (*p* = 0.027; medians: 0.61 for poor vs. 0.70 for good). A similar analysis within the subgroup of patients with squamous cell carcinoma and PD-L1 positivity (15 vs. 6) showed significant differences in maximum diameter (mm) (*p* = 0.004; means: 91,277 for poor vs. 58,821 for good; medians: 89,555 vs. 60,310); Sum_MBq (*p* = 0.011; means: 3.38 for poor vs. 0.927 for good); Hard_Area_Volume_cm3-Metabolic Tumor Volume (*p* = 0.014; medians: 116.255 for poor vs. 42.71 for good); Busyness (*p* = 0.029; medians: 0.69815 for poor vs. 0.60120 for good); and coarseness (*p* = 0.029; medians: 0.00835 for poor vs. 0.02530 for good). Conversely, the adenocarcinoma subgroup lacked a sufficient sample size for this analysis.

When comparing imaging parameters based on histological subtypes (squamous cell carcinoma vs. adenocarcinoma), significant differences were found in the following measures: SUV max (*p* = 0.034; mean for squamous vs. adeno: 20.047 vs. 15.197; median: 19.60 vs. 12.34); deviation (*p* = 0.047; median for squamous vs. adeno: 3.88 vs. 2.33); SUV mean (*p* = 0.035; median: 8.77 vs. 6.38); SUV Peak (*p* = 0.040; median: 16.96 vs. 11.12); and complexity (*p* = 0.045; median: 505.09 vs. 256.85).

We also examined the potential significant difference between PD-L1-positive and -negative groups (38 vs. 18). Analysis of the entire cohort, including both histological subtypes, showed no differences in imaging parameters. However, when focusing solely on the squamous cell carcinoma subgroup (29 patients, with 8 PD-L1-negative and 21 -positive), a significant difference was observed in the following parameters: Zone percentage (*p* = 0.039; mean: 0.299 vs. 0.199; median: 0.388 vs. 0.195 for PD-L1-negative vs. -positive) and long-zone high grey-level emphasis (*p* = 0.024; median: 830.11 vs. 1375.64). In the adenocarcinoma subgroup (17 PD-L1-positive and 10 -negative), no differences were identified in the parameters. Additionally, patients were grouped based on PD-L1 positivity rates of 1–49% and above 50%, but no significant differences were found.

Using the chi-square test, we investigated whether there was a significant association between NLR-based prognostic groups and lymph node positivity (including [^18^F]FDG uptake in mediastinal lymph nodes characteristic of metastasis). No significant association was found.

## 3. Discussion

In our retrospective study at a single center, we analyzed NSCLC patients by characterizing their staging using conventional and textural radiomic features derived from [^18^F]FDG PET/CT. These imaging parameters were obtained from primary lung nodules before tissue sampling. In the current molecular era, where targeted therapies and immunotherapies are more accessible, and next-generation sequencing (NGS) is becoming more widely used [20,21]. The importance of non-invasive imaging diagnostics and data analysis could become essential for selecting the best treatment. In NSCLC, several important molecular targets within the main histological subtypes can be targeted with therapy, including ALK rearrangements, EGFR, BRAF, or ROS-1 mutations, PD-L1 expression, and KRAS mutations [22,23,24,25,26].

Most current studies on PD-L1 positivity and using PET/CT radiomics have examined diverse patient groups. In contrast, the selection criteria used in this study produced a notably homogeneous patient population [15,27,28,29].

We add to the existing literature by focusing on a notably uniform group of PD-L1-positive patients. All of these patients received consolidation therapy with durvalumab after undergoing radio-chemotherapy.

[^18^F]FDG PET/CT remains the gold standard for precisely determining disease extent, lymph node involvement, and metastasis, while also guiding biopsies and tracking treatment response. Recent research indicates that [^18^F]FDG PET/CT might also predict molecular changes within lesions, which could enhance assessments of treatment efficacy and support personalized medicine approaches [30,31,32,33].

Firstly, consistent with other studies, we aimed to identify significant differences in imaging parameters between patient groups with PD-L1-positive and PD-L1-negative tumors. [^18^F]FDG PET/CT radiomic parameters of PD-L1-negative patients served as a control group. When analyzing both histological (adeno- and squamous) subtypes together, no significant differences in imaging features were observed between PD-L1-positive and PD-L1-negative cases. However, within the squamous cell carcinoma subgroup, several texture-based parameters showed significant differences between PD-L1-positive and PD-L1-negative patients. The median values of zone percentage and long-zone high grey level emphasis image features were notably higher in the negative group. Several previous studies have addressed this issue, which will be briefly discussed below. In a prior study [34], primary tumors were segmented on [^18^F]FDG PET/CT images of patients with non-small cell lung cancer, including adenocarcinoma and squamous cell carcinoma subtypes, to explore the potential for non-invasive assessment of PD-L1 status. In that study, a significant difference in SUVmax and SUVpeak was shown between PD-L1-positive and PD-L1-negative cases. However, no other clinical factors were assessed. Moreover, the patient population in the referenced article was heterogeneous in terms of disease stage [34]. In contrast, our analysis focuses only on stage III patients who received the same treatment protocol and were additionally stratified according to neutrophil-to-lymphocyte ratio (NLR) groups. Thereby we aimed to identify imaging-derived parameters that could serve as potential prognostic markers for future studies. A meta-analysis of a diverse patient group found that [^18^F]FDG PET/CT demonstrates only moderate sensitivity and specificity in assessing PD-L1 expression [35].

Other research has investigated alternative radiotracers for assessing PD-L1 positivity. For example, a study utilizing [^18^F]FAPI-04 PET/CT examined quantitative metrics in patients with histologically confirmed esophageal squamous cell carcinoma, comparing PD-L1-positive and PD-L1-negative groups. The results showed significantly elevated SUVmax, SUVmean, SUVpeak, and SUVsd values in PD-L1-positive, locally advanced cases [36]. Additionally, there is growing interest in using 89Zr-atezolizumab for precise lesion-based PD-L1 imaging and predicting patient outcomes [37]. However, this approach is not yet widely available, whereas deriving clinically valuable data from standard [^18^F]FDG PET/CT scans could provide a cost-effective and easily accessible alternative. Our findings suggest that the differences between PD-L1-positive and -negative lesions might be more significant in squamous cell lung carcinoma.

The two most common histological subtypes of NSCLC are adenocarcinoma and squamous cell carcinoma [1,38,39]. In this context, the patients in our cohort belonged to these two groups. Based on prior research, we hypothesized that these two main NSCLC subtypes could be distinguished using SUV-based metrics. Previous studies have indicated that squamous cell carcinomas usually show higher SUV values [40,41].

Our results align with previous findings, showing significant differences in SUVmax, SUVpeak, mean, deviation, and complexity between the two histological subtypes, with squamous cell carcinomas exhibiting higher median and mean values. Importantly, there was no significant difference in tumor volume or diameter between the groups, indicating that differences in SUV values are not related to tumor size. The same was observed when evaluating PD-L1 positivity.

Currently, testing for PD-L1 expression is the most crucial biomarker for predicting a patient’s chances of responding well to immunotherapies [42,43]. Since this is one of the most crucial data points influencing therapeutic decisions, it would be a breakthrough in patient care if we could provide similar decisive, non-invasive prognostic parameters using [^18^F]FDG PET/CT. Among laboratory tests, the neutrophil-to-lymphocyte ratio (NLR) stands out as particularly valuable in predicting how tumors respond to immune checkpoint inhibitors [17,18,19]. Its prognostic role has been previously studied in SCLC patients, comparing it with [^18^F]FDG PET/CT parameters. A previous study indicated that the strongest correlation was between NLR and MTV, WBMTV, and WBTLG [44]. We focused on PD-L1 expression and NLR values in our research because their combined prognostic significance has been analyzed in multiple other studies, showing promising results that support further investigation [45,46].

Therefore, we employed imaging metrics to compare NLR-based prognostic groups within the PD-L1-positive patient cohort. Our hypothesis was that this comparison could reveal whether imaging features offer prognostic information comparable to NLR, a validated predictive marker in PD-L1-positive non-small cell lung cancer (NSCLC). Similar studies, influencing perspectives, have been performed on more diverse groups, considering a broader range of patient data and clinical factors [47]. The metabolic–inflammation comprehensive prognostic index (MICPI) was evaluated alongside FDG-PET/CT parameters. NLR, a component of systemic inflammatory processes, is also considered among metabolic inflammatory factors. The study’s findings indicate that metabolic biomarkers may serve as useful tools for prognosis estimation [47].

In our retrospective analysis of the entire study population, we observed significant differences in parameters such as maximum diameter, short-zone emphasis, and zone-length non-uniformity. The metabolic tumor volume (MTV) approached significance (near *p* = 0.05), indicating it could achieve statistical significance with a larger sample size. In the subgroup of patients with squamous cell carcinoma and PD-L1 positivity, additional imaging parameters also demonstrated significant differences, including metabolic tumor volume (Hard_Area_Volume), busyness, and coarseness.

Previous studies have also shown that tumor diameter and metabolic tumor volume are key prognostic parameters in NSCLC and other types of tumors [48,49,50,51]. In invasive lung adenocarcinomas, the median SUVmax and entropy values were significantly higher in solid and micropapillary adenocarcinoma (SA-MA), while they were lower in lepidic adenocarcinoma (LA) [52]. A retrospective study examined the link between GLCM entropy measured by [^18^F]FDG PET/CT and the response to anti-PD-1/PD-L1 monotherapy in the initial trial for extensive-stage NSCLC, but did not find a significant difference [28].

Our findings indicate that, particularly in squamous cell carcinoma, [16]FDG PET parameters may play an even more significant role in evaluating PD-L1 positivity and in prognostic assessment. However, it is important to acknowledge certain limitations of our study. One of the main limitations of this study is its retrospective, single-center design and the relatively small sample size, particularly in certain subgroups. The smallest subgroup consisted of PD-L1-positive squamous cell carcinoma patients with poor NLR prognosis (n = 6). Consequently, subgroup analyses were performed in an exploratory, hypothesis-generating manner. Although statistically significant differences were observed, the limited statistical power warrants cautious interpretation of these findings. Furthermore, we cannot yet provide overall survival (OS) and progression-free survival (PFS) data, as most patients have only recently completed the final phase of their consolidation durvalumab therapy. We intend to update our results by incorporating these data in a future follow-up study.

It would also be beneficial to test AI-based prognostic models to enhance statistical robustness. However, their effectiveness may be more significant in larger groups—over 100 individuals for testing purposes. There are already existing studies involving deep learning and machine learning on this subject [29,48,53,54].

Furthermore, consistent with existing research, we recommend that the field pursue prospective studies to assess the prognostic and diagnostic utility of [16]FDG PET imaging metrics. Overall, our results highlight the necessity for further research in NSCLC to evaluate the importance of imaging biomarkers alongside histological and molecular characteristics. In the age of personalized medicine, advanced non-invasive diagnostic tools like PET/CT—particularly in NSCLC—can enable clinicians to manage their patients more effectively and precisely.

## 4. Materials and Methods

### 4.1. Patients

The patient cohort was chosen from the Clinical Center of the University of Pécs. All participants were treated in the Department of Oncotherapy and underwent diagnostic [^18^F]FDG-PET/CT scans at the Department of Medical Imaging, Nuclear Medicine Division in Pécs. The patients were inoperable, stage IIIA/IIIB, received radiochemotherapy as recommended, and then underwent immunotherapy depending on PD-L1 status. PD-L1-negative patients ineligible for immunotherapy served as controls. The study included two histological groups: squamous cell carcinoma and adenocarcinoma. Participants included men and women over 18 years old (average age 63.9 ± 5.9). Our objective was to select a homogeneous cohort under strictly uniform conditions.

### 4.2. Image Acquisition

PET and simultaneous low-dose CT images were acquired using the Mediso AnyScan 16 PET/CT scanner (Mediso, Budapest, Hungary). Before administering [^18^F]FDG (Pozitron Medical Kft., Budapest, Hungary), patients fasted for 6 h. Serum glucose levels were checked, and if the level exceeded 9 mmol/L, the imaging procedure was postponed. Acquisition began 60 min after intravenous injection of [^18^F]FDG with a mean activity of 3–4 MBq/kg. After the injection, patients rested in a darkened room. PET acquisition followed the CT scan. The 3D acquisition mode was used for PET data collection with a 3-min frame time. Depending on patient size, 7 to 9 bed positions covered the scan range, with an axial FOV of 15.12 cm (longitudinal FOV in the patient’s z-axis). The scans were corrected for scatter and randoms, and, according to the manufacturer’s recommendations, PET images were reconstructed iteratively using the Tera-Tomo™ 3D algorithm on a 167 × 167 × 234 matrix, resulting in an isotropic voxel size of 4 mm.

### 4.3. Segmentation, Imaging Parameter Extraction

**Figure 1:**
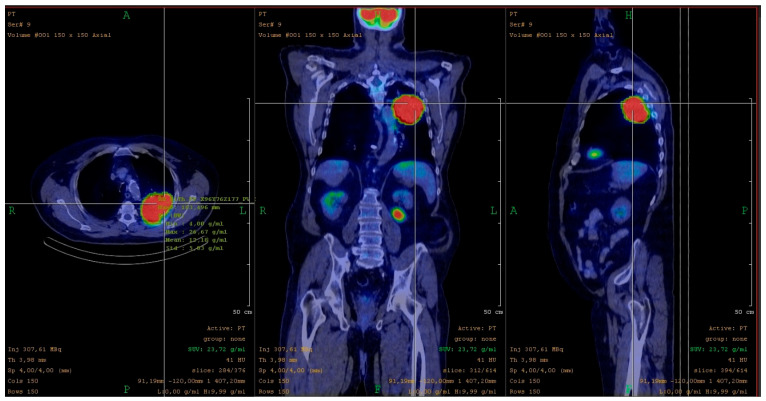
Segmentation of PD-L1-positive patient with adenocarcinoma.

After gathering the relevant clinical characteristics of the patients, the primary tumors were segmented with MEDISO Fusion software (version 3.12.002.0000). During segmentation, all measurable imaging parameters from the vendor were extracted and compiled into a comprehensive dataset. The SUVmax value of 4 g/mL served as the threshold for the semi-automated segmentation algorithm (see Figure 1). From the segmented VOI’s data, features such as intensity, histogram, morphology, neighborhood gray-tone difference matrix (NGTDM), gray-level co-occurrence matrix (GLCM), gray-level run length matrix (GLRLM), and gray-level size zone matrix (GLSZM) were extracted. Several previous studies have reported on radiomic analyses using this approach software [55,56,57].

### 4.4. Delineation and Feature Extraction

The target lesions were identified using InterViewTM FUSION clinical evaluation software. We set the SUVmax value of 4 g/mL as the reference threshold for the semi-automated segmentation algorithm. The SUVmax threshold of 4 g/mL was selected based on preliminary testing, as it allowed accurate tumor delineation without manual correction, ensuring reproducible and operator-independent segmentation. The software automatically generated key data for the delineated volume, including SUVmax, SUVmean, TLG, and tumor diameter.

### 4.5. Statistical Analysis

After extracting radiomic parameters, patient groups were categorized based on histology, PD-L1 status, and NLR-based prognosis. The neutrophil-to-lymphocyte ratio (NLR) was included in the analysis because, in the context of a retrospective study design, only already available and routinely collected clinical parameters could be reliably integrated into our research. All statistical analyses were conducted with Jamovi software. The Shapiro–Wilk test was used to assess the normality of continuous variable distributions. Variables with a normal distribution were analyzed using parametric tests, such as the independent samples *t*-test or one-way ANOVA. Non-normal variables were analyzed with non-parametric tests like the Mann–Whitney U test or Kruskal–Wallis test. A *p*-value of ≤0.05 was considered statistically significant. The chi-square test was employed to evaluate associations between two categorical variables.

## Figures and Tables

**Table 1:** Patients characteristics.

Variable	Number of Patients
n	56
Mean age (range)	63.9 (47–75)
Gender	
Male	30
Female	26
Clinical stage	
IIIA	32
IIIB	24
PD-L1 expression	
Positive	38
Negative	18
Histological type	
Squamous cell carcinoma	29
Adenocarcinoma	27
PD-L1-positive NLR status	
<5	30
>5	8
Lymph node involvement	42

## Data Availability

The original contributions presented in this study are included in the article. Further inquiries can be directed to the corresponding author.
